# Cyclopamine is a novel Hedgehog signaling inhibitor with significant anti-proliferative, anti-invasive and anti-estrogenic potency in human breast cancer cells

**DOI:** 10.3892/ol.2013.1195

**Published:** 2013-02-18

**Authors:** JUN CHE, FU-ZHENG ZHANG, CHAO-QIAN ZHAO, XU-DONG HU, SAI-JUN FAN

**Affiliations:** 1 Key Laboratory of Radiation Biology, School of Radiation Medicine and Protection, Medical College of Soochow University, Suzhou, Jiangsu 215123;; 2 Department of Radiation Oncology, The Fourth Hospital Affiliated to Soochow University, Wuxi, Jiangsu 214062, P.R. China

**Keywords:** cyclopamine, breast cancer, MAPK/ERK, estrogen receptor-α, proliferation, invasion

## Abstract

Stimulation of Hedgehog (Hh) signaling induces carcinogenesis or promotes cell survival in cancers of multiple organs. In epithelial cancer with aberrant Hedgehog activation, abrogation of Hedgehog signaling by cyclopamine, a naturally occurring Hedgehog-specific small-molecule inhibitor, causes profound inhibition of tumor growth. In the present study, cyclopamine displayed a significant potency in suppressing the proliferation of both estrogen-responsive (MCF-7) and estrogen-independent (MDA-MB-231) human breast cancer cells. Cyclopamine induced a robust G1 cell cycle arrest and elicited notable effects on the expression of cyclin D1 through modulation of the MAPK/ERK signaling pathway. Cyclopamine also inhibited the invasive ability of both breast cancer cell lines by suppressing the expression levels of NF-κB, MMP2 and MMP9 protein. Furthermore, in estrogen-responsive MCF-7 cells, cyclopamine significantly downregulated the production of estrogen receptor-α protein. Our results implicate cyclopamine as a novel, potent inhibitor of human breast cancer proliferation and estrogen responsiveness that could potentially be developed into a promising therapeutic agent for the treatment of breast cancer.

## Introduction

It was anticipated that by 2010, breast cancer would be newly diagnosed in >1.5 million females each year, and that 500,000 females worldwide would succumb to this disease (1). Therapies that target the drivers of individual types of breast cancer have substantially improved the outcome of females with breast cancer (2,3).

One such pathway is the hedgehog (Hh) signaling pathway, which specifies patterns of cell growth and differentiation during embryogenesis in a wide range of tissues (4). In addition to its role in developmental patterning, this pathway plays a critical role in mature tissue homeostasis and the maintenance of somatic cell numbers in various organs (5). Aberrant activation of the Hh pathway is common in breast carcinoma (6). The Hh pathway represents an attractive target for drug development and has demonstrated potential in clinical trials of cancer treatments. The specificity of cyclopamine for the Hh pathway is demonstrated by an absence of cytotoxicity in cells that lack Hh signaling (6).

In the present study, using estrogen-responsive (MCF-7) and estrogen-independent (MDA-MB-231) human breast cancer cell lines, the effects of cyclopamine on breast cancer proliferation and invasion are investigated *in vitro*. The possible pathways involving its regulation of breast cancer tumorigenesis and metastasis are explored.

## Materials and methods

### Cell culture and reagents

The MCF-7 and MDA-MB-231 human breast cancer cell lines were purchased from the American Type Culture Collection (Manassas, VA, USA). Cells were maintained in Dulbecco’s modified Eagle’s medium supplemented with 10% fetal calf serum, L-glutamine (5 mmol/l), non-essential amino acids (5 mmol/l), penicillin (100 U/ml) and streptomycin (100 U/ml) (Invitrogen Life Technologies, Carlsbad, CA, USA), at 37°C in a humidified atmosphere at 5% CO_2_. Cyclopamine and the MEK1/2 inhibitor, U0126, were obtained from Cell Signaling Technology, Inc. (Beverly, MA, USA).

The study was approved by the Ethics Committee of Soochow University, Suzhou, Jiangsu, China.

### Cell viability assay

Cell proliferation was determined using an MTT viability assay; the most commonly used assay for determining cell growth and death. The MTT survival assay has been described in detail previously (7). Exponentially growing cells were recultured (5,000 cells/well) overnight in 96-well tissue culture plates. Up to 20 *μ*l MTT (Sigma-Aldrich, St Louis, MO, USA) was directly added to the media in each well, at a final concentration of 2 mg/ml. Following 4 h of incubation, the medium containing MTT was discarded, and 120 *μ*l dimethyl sulfoxide was added for 10 min. The absorbance was measured using an enzyme-linked immunosorbent assay reader at 570 nm, with the absorbance at 630 nm as the background correction. The cell viability was expressed as the percentage of untreated controls. All experiments were performed at least three times.

### Proliferation assay

Cells were counted and plated at the same initial density on 6-well plates. They were then treated with 10 or 20 *μ*mol/l cyclopamine or the vehicle only, and incubated for time periods ranging from 0–10 days. At each time point, cells were trypsinized and counted using a Neubauer hemocytometer under trypan blue exclusion.

### Cell cycle assays

The cells were removed with trypsin and collected in centrifuge tubes together with the culture medium. The contents were centrifuged for 5 min at 1,800 × g. The supernatant was poured out, washed once with 1X phosphate-buffered saline (PBS) and centrifuged for a further 5 min. The cells were fixed with 5 ml of pre-cooled 70% ethanol for ≥4 h. The fixed cells were centrifuged and washed with 1X PBS. Following centrifugation, the cell pellets were resuspended in 500 *μ*l propidium iodide (10 *μ*g/ml) containing 300 *μ*g/ml RNase (Sigma-Aldrich). Subsequently, the cells were incubated on ice for 30 min, and then filtered with a 53-*μ*m nylon mesh. The cell cycle distribution was calculated from 10,000 cells with ModFit LT software (Becton Dickinson, San José, CA, USA) using FACSCalibur (Becton Dickinson).

### Transwell invasion assay

The invasion assay was carried out using Transwell plates (Millipore, Billerica, MA, USA), as previously described (8). The filter surfaces (pore size, 8 *μ*m) of the Transwell plates were uniformly coated with 25 mg Matrigel (Becton Dickinson, San Jose, CA, USA) overnight at 4°C, prior to the experiment. The lower chamber was filled with culture medium containing 10% fetal calf serum. The subconfluent proliferating cells were carefully transferred onto the coated upper surface of the chamber. Following 24 h of incubation, the filter was gently removed and the upper surface was wiped to remove all attached cells. The cells that had invaded through the Matrigel and attached to the lower surface of the filter were fixed with 4% paraformaldehyde and stained with Giemsa (Sigma-Aldrich, St Louis, MO, USA). Three replicates were conducted for each condition, and 15 random fields in each replicate were selected and counted using an Olympus CKX41 inverted microscope (Olympus, Tokyo, Japan). The results are presented as the ratio of the cells that invaded under experimental conditions relative to those that invaded under control conditions.

### Western blot analysis

Cell lysates were prepared and western blot analysis was performed as previously described (9). Equal aliquots of total cell protein (50 *μ*g/lane) were electrophoresed on sodium dodecyl sulfate (SDS)-polyacrylamide gels, transferred onto polyvinylidene fluoride (PVDF) membranes and then blotted using the following primary antibodies (1:1,000 dilution; Santa Cruz Biotechnology, Inc., Santa Cruz, CA, USA): β-actin (C-4), NF-κB (P65A), cyclin D1 (A-12), MMP2 (2C1), MMP9 (6-6B); along with secondary antibody horse-radish peroxidase-labeled goat anti-mouse (GAM-007) and goat anti-rabbit (SC-2004) IgG. The protein bands were visualized using an enhanced chemiluminescence system (Union Bioscience Corporation, Hangzhou, China) with prestained markers as molecular size standards. The densitometry of the protein bands was quantified with Quantity One (Bio-Rad, Hercules, CA, USA), and the values were expressed relative to β-actin (the control for loading and transfer). At least three independent experiments were performed for each cell type studied.

## Results

### Cyclopamine decreases breast cancer cell proliferation

**Figure 1:**
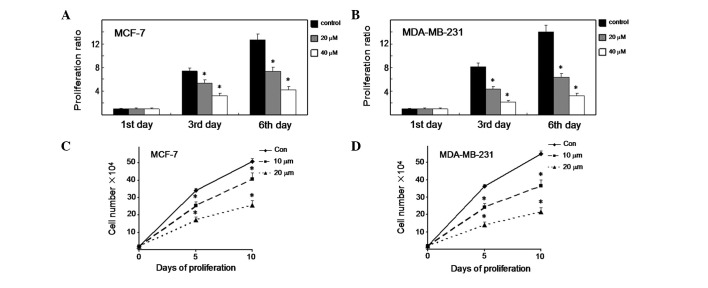
Cyclopamine decreases the proliferation of human breast cancer cells. (A and B) Breast cancer cells were treated with or without cyclopamine for 6 days, and then counted at the indicated time. All samples were prepared in triplicate. The proliferation rate was measured as fold changes in cell growth. (C and D) Proliferation curve of breast cancer cells. Cells were treated with cyclopamine and counted after 5 and 10 days. Results are representative of three independent experiments.

MTT viability assays were conducted to elucidate the potential biological effects of cyclopamine in breast cancer cells. As shown in Fig. 1A, the MCF-7 cells treated with cyclopamine displayed a significant reduction in proliferation rate compared with the control cells on days 3 and 6 (P<0.01). Significantly, cyclopamine demonstrated the same effect in MDA-MB-231 cells (Fig. 1B) (P<0.01). We also tested the effect of cyclopamine on cell proliferation using an alternative method. MCF-7 cells were treated with cyclopamine (10 and 20 *μ*M) or the vehicle only and incubated for time periods ranging from 0–10 days (Fig. 1C). At each time point, the cells were trypsinized and counted using a Neubauer hemocytometer under trypan blue exclusion. Cyclopamine was observed to induce a significant decrease in cell proliferation 5 and 10 days later (P<0.01). Notably, cyclopamine demonstrated the same effect in MDA-MB-231 cells (Fig. 1D) (P<0.01). The results imply that cyclopamine plays a key role in the growth control of breast cancer cells.

### Cyclopamine induces G1 cell cycle arrest and inhibits the invasive ability of both estrogen-responsive and non-responsive human breast cancer cells

**Figure 2:**
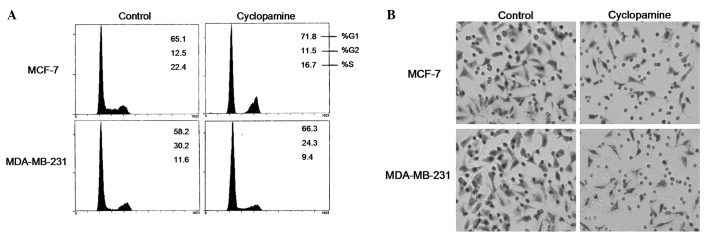
Cyclopamine affects breast cancer cell cycle and invasive ability. (A) Cell cycle distribution was evaluated by propidium iodide labeling and analysed by flow cytometry. (B) Cells treated with or without cyclopamine were trypsinized and transferred to the upper compartment of the modified Transwell chambers. Following 24 h of incubation, the invasive cells attached to the lower surface of the Matrigel-coated filter were fixed, stained and photographed under a phase contrast microscope and then counted in 15 randomly selected microscopic fields.

Cell cycle analysis was then performed on the regulation of cyclopamine in breast cancer cells. The results in Fig. 2A suggest that cyclopamine significantly induced cell accumulation in the G1 phase (P<0.01) and a modest decrease in the S population percentage, from 22 to 16% (P<0.01) in the MCF-7 cells. Moreover, cyclopamine also caused a significant increase in G1 cells in the MDA-MB-231 cells.

A critical event in tumor metastasis and progression is the ability of tumor cells to invade the extracellular matrix, allowing the tumor cells to move beyond the restrictions of the primary tumor environment. To examine the competency of cells to invade through biological matrices *in vitro*, a Transwell assay was conducted as described previously. The results demonstrated that, compared with the control cells, cyclopamine vigorously inhibited the ability of the MCF-7 and MDA-MB-231 cells to invade through the filter coated with Matrigel. As shown in Fig. 2B, the invasion rate of cyclopamine-treated cells significantly decreased compared with the control cells.

### Cyclopamine affected the expression level of cell cycle- and invasion-related proteins

**Figure 3:**
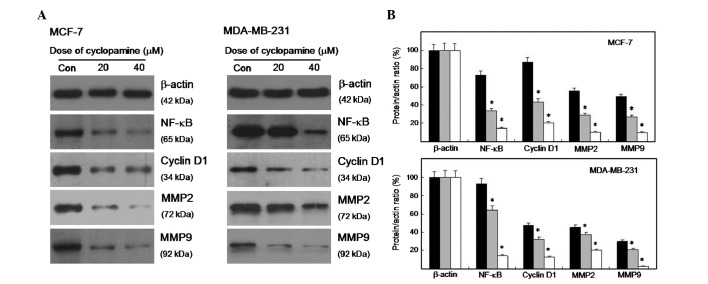
Cyclopamine inhibits the expression of proliferation and invasion-related proteins. (A) Whole cell lysates were prepared, and 50 *μ*g proteins were resolved using SDS-PAGE, followed by immunoblotting with the indicated specific antibodies against NF-κB, cyclin D1, MMP2 and MMP9. (B) The expression levels are displayed as fold changes in band density. ^*^P<0.01 vs. the control group.

The alterations in the cell cycle- and invasion-related proteins in cyclopamine-treated cells were evaluated and compared with the control cells using western blot analysis. Fig. 3A shows that the expression level of cyclin D1 decreased in cyclopamine-treated cells. The expression levels of NF-κB, MMP2 and MMP9 were also suppressed. The densitometry of the protein bands was quantified with Quantity One, and the values were expressed relative to β-actin. As shown in Fig. 3B, the expression level of the four proteins significantly decreased (P<0.01).

### Cyclopamine modulates the expression of cyclin D1, via the MAPK/ERK signaling pathway, and downregulates the expression of ER-α

**Figure 4:**
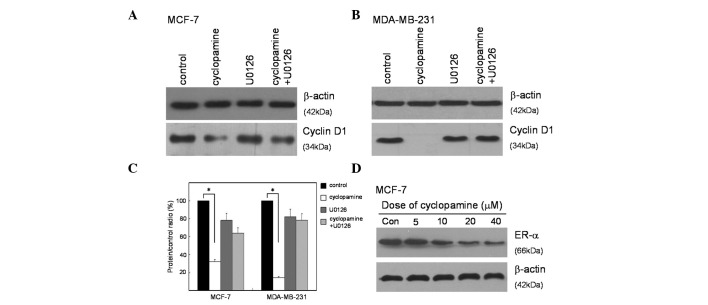
U0126 partly rescues cyclin D1 inhibition to cyclopamine treatment. (A and B) Comparison of cyclin D1 expression between cells treated with or without cyclopamine. (C) Statistical analysis was conducted using a Student’s t-test. The expression levels are displayed as fold changes in band density. ^*^P<0.01 vs. the control group. (D) Dose-dependent effects of cyclopamine on expression of estrogen receptor-α (ER-α) protein. MCF-7 cells were treated with indicated concentrations of cyclopamine for 24 h, and the level of ER-α was determined by western blot analysis.

Western blot analysis was utilized to identify the targets of the Hedgehog signal pathway that may be involved in suppressing proliferation. The expression of cyclin D1 is a key initial response to cell cycle distribution and proliferation. The specific signaling cascade involved in this response was explored using a MEK1/2 inhibitor (U0126) specific to the MAPK/ERK pathway. The results showed that cyclopamine significantly inhibited the expression of cyclin D1 in both MCF-7 and MDA-MB-231 cells. To certify the potential effect of MAPK/ERK in response to cyclopamine treatment, the two cell lines were treated with U0126 prior to cyclopamine treatment. The results demonstrated that U0126 could partially prevent cells from cyclopamine-induced cyclin D1 inhibition (Fig.4A and B). The reduced cyclin D1 expression in cyclopamine-treated cells was inhibited by the MEK1/2 inhibitor (Fig. 4C), which suggested that cyclopamine mediated the expression of cyclin D1 through modulating the MAPK/ERK pathway.

The underlying mechanism for the ability of the majority of anticancer drugs to cause a growth arrest of breast cancer, is that the drugs downregulate the expression of ER-α. To determine whether cyclopamine has a similar effect on ER-α production, MCF-7 human breast cancer cells were treated with a range of concentrations of cyclopamine and the level of total ER-α protein was monitored by western blot analysis of total cell extracts. As shown in Fig. 4D, cyclopamine strongly downregulated ER-α levels, and β-actin was used as a constitutive gel loading control.

## Disscusion

Breast cancer is the second leading cause of cancer-related mortality in females worldwide. Alternative strategies are required to combat the deaths caused by this disease. Chemotherapy, radiation, surgery and immunotherapy are among the current treatment options for breast cancer. Chemotherapy using synthetic compounds, although demonstrated to be effective in cancer treatment, also induces severe side effects due to their toxicity in non-cancerous tissues (10). In recent years, therapeutic strategies that specifically target aberrant signaling pathways in metastatic breast cancer greatly enhance survival, while at the same time reducing bystander toxicity in non-tumor tissues (11).

The MAPK pathway plays an important role in regulating a number of downstream molecules including kinases and scaffold proteins (12). The balance between these molecules exerts cellular responses, including cell proliferation, cell cycle arrest, migration and differentiation. Extracellular signal-regulated kinases are members of the MAPK family that transduce signals from various environmental stresses, growth factors and steroid hormones (13).

The present study has identified that cyclopamine has potent antiproliferative properties as a potential therapeutic agent for the treatment of human breast cancers by suppressing MAPK/ERK-mediated signaling. Our results show that cyclopamine significantly increased the potency of the cell cycle arrest in both human estrogen-responsive and estrogen-independent human breast cancer cells, which suggests that it may potentially be used for treating a wide range of breast cancer types, which has been a fundamental problem with the available therapeutic compounds.

Furthermore, cyclopamine inhibits the expression of ER-α, which mediates the mitogenic properties of estrogens, and acts with tamoxifen to inhibit the proliferation of steroid-responsive MCF-7 human breast cancer cells to a greater extent than either compound alone. This result implicates the use of cyclopamine in combination with anti-estrogen therapies that would allow lower doses and thereby reduced side effects or decreased resistance to the anti-estrogens.
